# Molecular genetic testing of congenital adrenal hyperplasia due to 21-hydroxylase deficiency should include CAH-X chimeras

**DOI:** 10.1038/s41431-021-00870-5

**Published:** 2021-04-07

**Authors:** Qizong Lao, Deborah P. Merke

**Affiliations:** 1 Section on Congenital Disorders, National Institutes of Health Clinical Center, Bethesda, MD USA; 2 Eunice Kennedy Shriver National Institute of Child Health and Human Development, Bethesda, MD USA

**Keywords:** Genetic testing, Adrenal gland diseases

## To the Editor:

We read with great interest the European Molecular Genetics Quality Network practice guidelines for molecular genetic testing of congenital adrenal hyperplasia (CAH) due to *CYP21A2* defects [1]. The authors address multiple important issues in the molecular genetic testing of CAH and provide valuable suggested standards based on extensive experience and practices from laboratories worldwide. While in general we agree with the workflow and methods described in the guidelines, we appeal to include evaluation for a contiguous gene deletion syndrome, termed CAH-X, within the scope of the testing. We believe this would be particularly beneficial for individuals carrying a CAH genotype of “30 kb deletion”.

As illustrated in Fig. 2 of the article by Baumgartner-Parzer et al. [1], the terms “30 kb deletion” or “large gene conversion” refer to a CAH genotype of chimeric genes caused by unequal crossover during meiosis due to the structural complexity of the *CYP21A2* locus. These deletions account for 20–30% of all CAH alleles and are categorized into two subtypes determined by the location of the junction sites: CAH and CAH-X chimeras [2]. CAH chimeras (*CYP21A1P/CYP21A2*) are caused by recombination between *CYP21A1P* and *CYP21A2*, and impair only *CYP21A2*; CAH-X chimeras (*CYP21A1P-TNXA/TNXB)* are caused by recombination between *TNXA* and *TNXB*, null the entire *CYP21A2* and replace the 3′ of neighboring *TNXB* with the pseudogene *TNXA*. This contiguous deletion is hence termed CAH-X, named for both *CYP21A2* and *TNXB* defects. The latter is causative for hypermobility type Ehlers–Danlos syndrome (EDS) due to either deficiency or misfolding of its encoding tenascin-X, a crucial component of the extracellular matrix [3–5].

Unlike the well documented CAH chimeras, CAH-X chimeras have often been missed and are typically omitted from the scope of CAH testing, even though this contiguous gene deletion was first reported in 1997 [3]. As a result, CAH-X cases are reported as having a null CAH allele leading to lack of recognition and underestimation of CAH-X prevalence. In fact, the prevalence of CAH-X was recently estimated to be 15% in our large CAH cohort (278 subjects) in the U.S. followed at the National Institutes of Health Clinical Center (Bethesda, Maryland) and it was particularly prevalent (30%) in the “30 kb deletion” alleles [6]. Similarly, Gao et al. reported a 14% CAH-X prevalence in a large Chinese cohort (424 subjects) and a very high (59%) prevalence in CAH alleles previously reported as “deletion” or “null” [7]. These reports indicate the necessity of revisiting the prevalence of CAH-X chimeras and addressing potential clinical implications of the CAH-X syndrome, especially in subjects carrying an allele of “30 kb deletion” or “large gene conversion”.

CAH-X syndrome is composed of two elements: CAH and hypermobility type EDS; the former is autosomal recessive but the latter appears to be autosomal dominant. If carrying a CAH-X allele, phenotypic EDS is expected regardless of the CAH status [2], although in general CAH patients have more severe EDS manifestations than their carrier counterparts [4, 5]. The hypermobility EDS spectrum includes joint hypermobility, arthralgias, frequent joint dislocations, hernias and midline defects including cardiac structural abnormalities. Overall, about 25% of reported CAH-X patients have cardiac abnormalities including congenital heart defects such as structural valve abnormalities, left ventricular diverticulum, and patent foramen ovale [8]. To date, the clinical diagnosis of EDS in CAH-X mostly relies on evaluation of joint hypermobility and subluxations, which are often restricted by factors such as age and time of onset. Genetic testing for this germline condition in newborn screening is advantageous in terms of offering very early diagnosis to young children before hypermobility evaluation is applicable, and to enable early screening for cardiac defects. In older individuals undergoing *CYP21A2* genotyping, identification of CAH-X would provide information regarding the cause of any ongoing connective tissue abnormalities and guidance regarding preventative measures. Treatment for hypermobility type EDS depends on the signs and symptoms present, and a screening echocardiogram should be considered.

To date, most CAH genetic test platforms determine the status of “30 kb deletions”. Adding a CAH-X test selectively to “30 kb deletion” positives can be a pinpoint add-on. Sharing a ride with established DNA extraction and even PCR would optimize laboratory efficiency and cost-effectiveness. For instance, laboratories that use a classic CYP779f/tena32F primer pair for PCR can use the same PCR product to conduct Sanger sequencing tests of CAH variants, 30-kb deletions and CAH-X chimeras [9, 10]. A high throughput CAH-X screening has been developed, which has shown excellent accuracy. It is amenable to either qPCR or digital PCR, thus it is fast and easy to operate [6]. If MPLA methodology is used, an up-to-date version now simultaneously checks “30 kb deletion” and a CAH-X chimera of haploinsufficiency [1, 7], therefore labs would only need to test the CAH-X chimeras of misfolding on “30 kb deletion” positives to complete the CAH-X test by either Sanger or qPCR/digital PCR methodologies.

In summary, CAH-X chimeras account for a large portion of “30 kb deletion” CAH alleles and have historically been under-detected. Regardless of their CAH status, individuals who carry a CAH-X allele are expected to suffer from a spectrum of hypermobility EDS manifestations, including cardiac structural defects, joint hypermobility and instability, hernias and gastrointestinal disorders. Including CAH-X determination in CAH molecular genetic testing platforms is convenient and cost-effective, and is particularly beneficial for individuals carrying a “30 kb deletion” allele because many of them are in fact CAH-X. An early diagnosis of CAH-X syndrome is valuable for preventative care and long-term medical management.
